# Work-related stress predicted future sick leave in primary health care patients

**DOI:** 10.1093/eurpub/ckac131.265

**Published:** 2022-10-25

**Authors:** A-M Hultén, P Bjerkeli, K Holmgren

**Affiliations:** 1 Institute of Neuroscience and Physiology, University of Gothenburg, Gothenburg, Sweden; 2 Department for Public Health Research, University of Skövde, Skövde, Sweden

## Abstract

**Background:**

Studying the relationship between work-related stress and sick leave is valuable in taking actions for workers’ health. This study aimed to analyse the association between work-related stress, measured with the Work Stress Questionnaire (WSQ), and registered sick leave among primary health care patients in Sweden.

**Methods:**

The prospective longitudinal study included 232 patients who were non-sick-listed, employed, aged 18-64 years and sought care for mental and/or physical health complaints. Logistic regression analysis was performed with questionnaire data on work-related stress from baseline together with sick leave data from a national register for the following 12 months.

**Results:**

High stress due to indistinct organization and conflicts was reported by 21% (n = 49), while 45% (n = 105) reported high stress due to individual demands and commitment. During 12 months 36% (n = 83) were on sick leave for 15 days or more. The odds of being on sick leave was twice as high for patients perceiving high stress due to indistinct organization and conflicts, high stress due to individual demands and commitment, low influence at work, or high interference between work and leisure time. Perceiving high stress due to both indistinct organization and conflicts as well as individual demands and commitment quadrupled the odds of sick leave, OR 4.15 (95% CI 1.84; 9.38).

**Conclusions:**

Work-related stress and sick leave were prevalent among the patients. Primary health care can therefore be a suitable arena for addressing these issues. Perceiving work-related stressors and stress within one or multiple areas increased the odds of registered sick leave by two to four times. Hence, a wide spectrum of factors needs to be considered, to capture the dynamic interaction between the individual and the work environment.

**Key messages:**

• Work-related stress is associated with future sick leave for primary health care patients.

• Early identification of patients with work-related stress is important for the primary health care.

